# The dysfunction of Tfh cells promotes pediatric recurrent respiratory tract infections development by interfering humoral immune responses

**DOI:** 10.1016/j.heliyon.2023.e20778

**Published:** 2023-10-06

**Authors:** Jun Diao, Huosheng Liu, Hui Cao, Weibin Chen

**Affiliations:** a Department of Pediatrics, Yueyang Hospital of Chinese Integrative Medicine, Shanghai University of Traditional Chinese Medicine, Shanghai, China; b Department of Acupuncture and Moxibustion, Jiading Hospital of Traditional Chinese Medicine, Shanghai, 201800, China; c Department of Liver Diseases, Longhua Hospital, Shanghai University of Traditional Chinese Medicine, Shanghai, China

**Keywords:** Pediatric RRTIs, Humoral immunity, Tfh cells, Pathogenesis

## Abstract

Recurrent respiratory tract infections (RRTIs) are one of the most common pediatric diseases. Although the pathogenesis of pediatric RRTIs remains unknown, ineffective B cell-dominated humoral immunity has been considered as the core mechanism. During the course of pediatric RRTIs, B cell-dominated humoral immunity has changed from “protector” of respiratory system to “bystander” of respiratory tract infections. Under physiological condition, Tfh cells are essential for B cell-dominated humoral immunity, including regulating GC formation, promoting memory B cell (MB)/plasma cell (PC) differentiation, inducting immunoglobulin (Ig) class switching, and selecting affinity-matured antibodies. However, in disease states, Tfh cells are dysfunctional, which can be reflected by phenotypes and cytokine production. Tfh cell dysfunctions can cause the disorders of B cell-dominated humoral immunity, such as promoting B cell presented apoptosis, abrogating total Ig production, reducing MB/PC populations, and delaying affinity maturation of antigens-specific antibodies. In this review, we focused on the functions of B and Tfh cells in the homeostasis of respiratory system, and specifically discussed the disorders of humoral immunity and aberrant Tfh cell responses in the disease process of pediatric RRTIs. We hoped to provide some clues for the prevention and treatment of pediatric RRTIs.

## 1 Introduction

Recurrent respiratory tract infections (RRTIs) are one of the most common pediatric diseases [1]. The most widely accepted definition of pediatric RRTIs are as follows: eight or more illnesses due to respiratory infections per year in children <3 years, or six or more per year in children> 3 years [2]. Although each country or/and region has a slightly different of diagnostic criteria, the main characteristics of pediatric RRTIs are presenting by upper or/and lower respiratory tract infections that occur frequently per year. According to epidemiologic statistics, RRTIs are accounting for 10%–30 % of all pediatric respiratory infections, and have become a global disease with accelerating incidence in newly industrialized countries [3]. Although RRTIs are still a risk factor for the growth and development of children [4], the pathogenesis of this disease remains incompletely clarified. Accumulating evidences have demonstrated that B cell dysfunctions and immunoglobulin deficiencies positively correlated with the frequencies of respiratory infection in children with RRTIs [5,6]. Interestingly, B cells and immunoglobulins are considered as the hallmark of humoral immunity. Hence, the inefficiency of humoral immune responses may account for pediatric RRTIs development.

Mechanistically, the core functions of humoral immunity in the respiratory system are against pathogens invasion, which B cell immune responses are indispensable [7]. After pathogens invaded in respiratory system, B cells are activated, which ultimately differentiate into plasma cells (PCs) and memory B cells (MBs), both of them can prevent pathogens infections/reinfections [8] Noteworthy, in the process of B cell activation and differentiation, normalization of follicular helper T (Tfh) cell responses are absolutely necessary [9]. Undoubtedly, Tfh cell dysfunctions can dampen B cell programs, which weakened humoral immunity and increased incident of respiratory tract infections [10,11]. In the lesion location and peripheral blood (PB) of children with RRTIs, Tfh cell dysfunctions have been detected, and they aberrant responses were found to associated with disease progression [5]. However, the relationship between Tfh cell dysfunctions and humoral immunity is rarely reported in the pediatric RRTIs. Hence, this review focused on the defensive functions of humoral immunity in the respiratory system of children, and specifically discussed the abnormal B cell-dominated humoral immunity and the aberrant Tfh cell responses in the disease process of pediatric RRTIs. We hoped to provide some clues for exploring disease pathogenesis and drug development, which will be beneficial to pediatric RRTIs prevention and treatment.

### 1.1 The humoral immune responses in the respiratory system of children

It is generally thought that productive humoral immune responses require that naïve B cells and their differentiated progeny move among distinct micro-environments [12]. The development and differentiation and activation of B cells mainly proceed in the bone marrow and the peripheral lymphoid tissues [13]. In the bone marrow, lymphoid progenitor cells eventually differentiated into immature B cells after encountered the B cell receptor (BCR) functional rearrangement and positive and negative selection steps. After developed, immature B cells are licensed to traffic to peripheral lymphoid tissues in which they will develop into mature naïve B cells [14]. Unlike immature B cells that only express membrane immunoglobulin (mIg) M, mature naïve B cells can co-express mIgM and mIgD, and they recirculate through peripheral lymphoid tissues until encounter their cognate antigens [15]. Once perceive the appropriate antigens, mature naïve B cells rapidly initiate immune responses against pathogens by differentiate into long-live d MBs or antibody-secreting PCs [14].

**Fig. 1:**
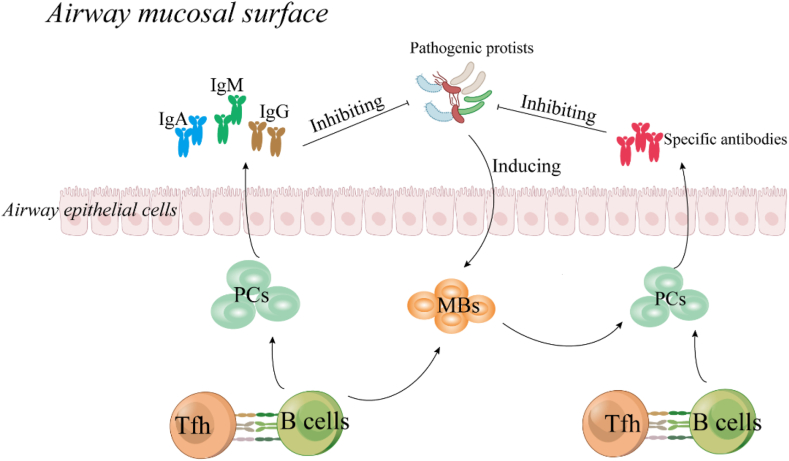
The biological functions of B cells in the airway mucosal surface. Under physiological condition, Tfh cells can support mature naïve B cells to differentiate into memory B cells (MBs) and plasma cells (PCs).PCs can prevent pathogens invasion by producing immunoglobulins (Igs) and specific antibodies. MBs can sense pathogen reinfections, and quickly eliminate pathogens by differentiating into PCs.

**Fig. 2:**
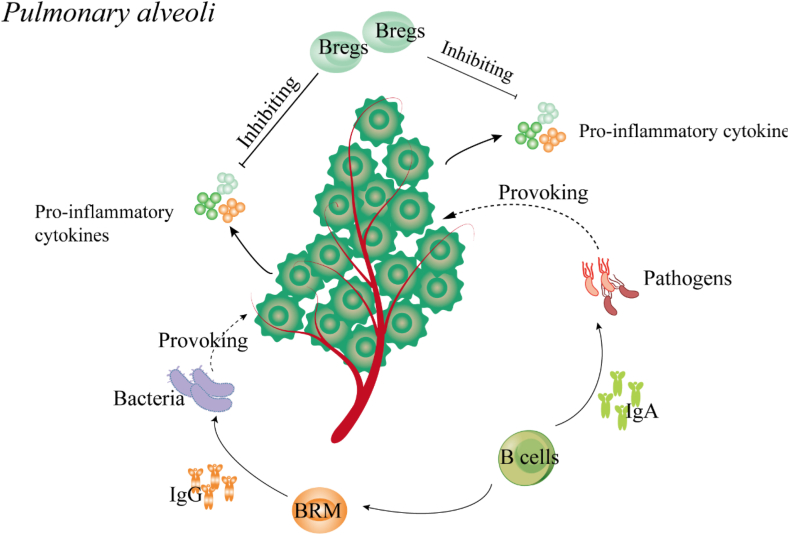
The critical roles of B cell populations in the pulmonary alveoli. B cell populations have three ways to against pathogens infections. At the first level, B cell populations (residing in the lung) can directly inhibit respiratory virus infections by secreting protective mucosal antiviral IgA. Secondly, lung-resident memory B cells (BRM) can provide to antibacterial immunity by secretion of heterotypic antipneumococcal IgG. More importantly, lung-resident regulatory B (Breg) cells can alleviate excessive lung inflammation by suppressing pro-inflammatory cytokines over-production via producing IL-10.

In general, in normal physiologic state, B cell distributions are significant differences according to the anatomic sites and age [16]. In the respiratory system of children, B cell s can be detected in the nasal associated lymphoid tissue, Waldeyer's ring (in the upper respiratory tract), and bronchial-associated lymphoid tissue (in the lower respiratory tract) [17]. In those secondary lymphoid organs (SLOs), following primary infection, B cells subsequently mature the affinity of their antibodies through somatic hypermutation and selection in germinal centers (GCs) [18]. The majority of MBs emerge from this pathway and join naive B cells that recirculate between those SLOs, continuously scanning the respiratory system for secondary infection [19]. Moreover, during primary infection, GCs-derived PCs also develop. PCs may migrate to the bone marrow where they constitutively secrete antibodies to prevent reinfections [20]. Classically, in the respiratory system, the primary biological function of B cells is producing immunoglobulins (Igs), such as IgA, IgG, IgD, IgE and IgM [21]. Those Igs distribute in the parenchyma as well as the mucosal surface of the airway, which can inhibit incoming pathogens attachment/entry via complement fixation and antibody-dependent cellular phagocytesis and cytotoxicity [22] (Fig. 1). The second critical role of B cells for respiratory system is mediating local immune responses during the pathogens infections (Fig. 2). Recent studies have shown that B cells that populated in the respiratory system are involved in preventing pathogens invasion. Such as, B cell populations of the respiratory mucosa can defense respiratory virus infections by secreting protective mucosal antiviral IgA [22]; lung-resident memory B cells (BRM cells) not only can provide to antibacterial immunity by secretion of heterotypic antipneumococcal IgG, but also can contribute to improving vaccine effectiveness [23,24]; lung-resident regulatory B (Breg) cells can alleviate excessive lung inflammation by suppressing pro-inflammatory cytokines over-production via producing IL-10 during pathogens infections [24,25]. Therefore, the normalization of B cell programs is indispensable for the homeostasis of respiratory system of children.

### 1.2 Impairment of humoral immunity is associated with pediatric RRTI development

It has been confirmed that ineffective humoral immune responses are associated with the occurrence of various pediatric diseases, and pediatric RRTIs is one of them [14,26]. While previous studies proposed that children with RRTIs have no significant alterations in their immunity, new evidences showed that their humoral immunity is impaired [27]. Indeed, in clinical practice, various pharmacological interventions are effective in treatment of pediatric RRTIs by regulating humoral immune responses [28,29]. As mentioned above, B cells dominated humoral immunity. Hence, abnormalities of their responses to pathogens are a sign of humoral immunity impairment in children with RRTIs. During the course of this disease, ineffective humoral immune responses can be reflected by the deficiency of Igs and PCs and MBs, all of them are related to the prevention of pathogens infections/reinfections [29]. Due to easy to detect, the Igs deficiency is the most frequently reported in pediatric RRTIs [30]. As Gerard Pasternak and colleagues have shown that the levels of IgG, IgM, and IgA in the serum of children with RRTIs were significantly lower than healthy children (HC) [31]. The similar result was proposed by Filome na Monica Cavaliere and colleagues, and they even found that serum titers of pathogens-specific Igs in children with RRTIs were still significantly lower than in age-matched controls after received vaccination [30]. As reported elsewhere, Igs are important players in the homeostasis of respiratory tract [32,33]. Hence, Igs deficiency indicated that humoral immunity of children with RRTIs has a defective response to pathogens invasion. Traditionally, Igs can be divided into five subtypes, including IgG, IgM, IgE, IgD, and IgA [34]. As the majority of serum Igs, IgG is essential for respiratory tract against bacterial invasion. Children who have higher levels of IgG are less susceptible to various respire tory infectious diseases [35]. Human IgG has two pathways for eliminating bacteria that invaded into peripheral circulation. One is binding to surface proteins of intact bacteria, which can promote their aggregation [35,36]. The other is binding to C1q to activate the complement system, which can contribute to opsonophagocytotic killing by depositing on the bacteria surfaces [37]. Except for the similar roles with IgG in defensing bacteria, IgM is also indispensable for preventing to pulmonary fungal infection in children, because IgM can recognize fungal proteins, which prime T cell responses [38]. Compared with IgG and IgM, IgA is more abundant in the healthy human respiratory tract, which is essential for lung defense against infection, particularly in children [39]. In the respiratory tracts of children, IgA can sequestrate endogenous bacteria by Fab-mediated antigen-specific binding, which limits the adherence and penetration of bacteria to the epithelial cells [40]. Moreover, IgA also can activate phagocytic system by binding to FcRα receptor, which is beneficial to the elimination of pathogenic antigens without inducing inflammation [39]. Noteworthy, IgA deficiency has been confirmed to be related to the progression of pediatric RRTIs [33,40].

In addition to Igs deficiency, the abnormal differentiation and activation of B cells are also the symbol of humoral immunity impairment in pediatric RRTIs. In recent years, some studies have confirmed that mature/naive B cell proportion in the peripheral circulation of children with RRTIs were significantly lower than that in HC [41]. As discussed above, mature/naive B cells can differentiate into MBs or/and PCs upon meet their specific antigens. Hence, there is no doubt that children with RRTIs also have fewer MBs and PCs compared with HC. However, although some scholars have found that the frequencies of MBs and PCs in children with RRTIs has not decreased, and ma y have even increased, subsequent evidences have proposed that those cells are functionally immature and abnormal [20,42]. MBs and PCs are critical for protection against pathogen infections/reinfections. Upon encounter to pathogen invasion, B cells are activated, and they will differentiate into PCs and MBs. PCs can inhibit pathogen proliferation by synthesizing and secreting massive amounts of antibodies (Ab) or Igs [43]. MBs not only can rapidly capture pathogen by secreting pathogens-specific antibodies, but also can against challenge by related pathogens or variant pathogens, which have escaped from PCs-mediated barrier [19,44,45]. The most prevalent pathogens that causing recurrent respiratory infections in children are *respiratory syncytial virus* (RSV), *human rhinovirus*, and *influenza virus* [46]. Despite the mechanism of persistent infections by those pathogens remains unclear, the dysfunction of resident B cells in the respiratory system has attracted considerable attention. As the B cells with anti-inflammatory properties in the respiratory system, abnormal responses of Breg cells can be observed in children with RRTIs [47]. During the course of the disease, the frequencies of Breg cells were increased, and they will secrete to excessive IL-10 via BCR-CX3CR1 pathway [48]. However, excessive IL-10 can significant dampen Th1 cytokines production, which prolonged the course of pathogens infections [49]. Indeed, an increased IL-10 release with decreased levels of Th1 cytokines were found in the children with RRTIs [50]. In addition, some scholars have proposed that Breg cells from nasal turbinates and lungs of RSV-infected mice are highly permissive to pathogens infection [50]. Similar to Breg cells, the responses of resident MBs to pathogens in children with RRTIs were also aberrant. Normally, in the face of pathogen invasion, adenoid resident IgM^+^ and/or IgD^+^ MBs in HC showed higher binding affinities and neutralization potencies, while their responses were horribly inefficient in children with RRTIs [[51], [52], [53], [54]]. Notable, although B cell dysfunctions can be detected in the course of pediatric RRTIs, there is no clear mechanisms to explain how B cells change from “protector” of respiratory system to “bystander” of respiratory tract infections. In the past few years, many studies have found that B cell dysfunctions in the children with RRTIs were accompanied by T cell aberration, especially for Tfh cells ^[ 5,11]^. Due to Tfh cells are mainly responsible for B cell programs, they have become an important breakthrough for rescuing B cell functions in pediatric RRTIs.Tfh cells are indispensable for humoral immune responses.

Compared with Th1, Th2 and Th17 cells, Tfh cells were found fairly later. The disco very of Tfh cells was resulted from the detection of CD4^+^T expressing CXCR5 in the secondary lymphoid organs [9]. With the identification of differentiation related pathway and specific transcription factors, in 2009, Tfh cells were eventually recognized as an independent CD4^+^T cell subsets [55]. The differentiation of Tfh cells is associated with the activation of STAT3 and STAT4, both of them can be triggered by dendritic cells (DCs)-derived cytokines [56]. After STAT3 and STAT4 activation, *Bcl-6* are transcribed and translated into Bcl-6, which indicated the beginning of Tfh cell differentiation and maturation [57]. After maturation, Tfh cells can secret various cytokines, including IL-21, IL-4, IL-9 and B-cell activating factor (BAFF) [58]. Similar to other CD4^+^T cell sub sets, the differentiation and activation of Tfh cells also require cytokine signals, such as IL-6, IL-12,TGF-β, and IL-27 [59]. After stimulation with those cytokines, the expression of multiple Tfh cell-related molecules can be up-regulated in the human naïve CD4^+^ Th cells, including CXCR5, ICOS, PD-1,CD40, and Bcl-6 [59]. Apparently, those molecules are critical for Tfh cell development and migration and functions, and also can reflect to their phenotypes. In general, Tfh cell phenotypes are related to where it is distributed [60]. In the lymphoid organs, germinal centers (GCs) Tfh cells express high levels of CXCR5 and ICOS, while those molecules are slight expressed in Tfh cells that are excessively localized outside GC [55]. In the peripheral blood (PB) of human, although the circulating Tfh cells have several populations with unique phenotypes, CXCR5^+^CD4^+^T cells are currently named as circulating Tfh cells [61]. Interestingly, unlike lymphoid organs Tfh cells, circulating Tfh cells can express the proliferation marker Ki67, which may imply that their clonotypic relationship is uncorrelated with lymphoid organs Tfh cells [61]. However, in a recent study, Elena Brenna and colleague found that clonotypic overlap between PB and lymphoid Tfh cell populations by analyzing their TCR repertoire, and then they proposed that Tfh cells in the PB have similar characteristics as lymphoid Tfh cells [62].

As reported elsewhere, Tfh cells are the CD4^+^T cells that specialized for helping B cells [9]. They play a critical role in supporting B cell immune responses, including regulation of GCs formation, promotion of MB/PC differentiation, induction of Ig class switching, and selection of affinity-matured antibodies [9,63]. Tfh cells mediate their effects via receptor-ligand interactions with B cells and production of cytokines (Table 1). As a central hallmark of Tfh cells, CXCR5 can traffic them migrate to the B-cell follicles, thereby promote T-B interactions. In addition, CXCR5 is also necessary for GC formation. GC development is impaired in the CXCR5^−/−^mice, which resulted in a significant reduction of GC B cells and isotype-switched antibody-secreting B cells and high-affinity antigen-specific IgG1 [60,62]. Like to CXCR5, ICOS deficiency in Tfh cells also contributes to severely damage of GCs. Moreover, Tfh cells with ICOS deficiency fail to express CXCR5 normally, which made them migrate into B cell follicles inefficiently [64]. As a co-stimulatory molecule, ICOS is also imperative for Tfh cell delivering functions [65,66]. Once Tfh cell activation, their destiny is residing in the follicles through crossing T-B border, and subsequently interact with GC B cells and provide help signals via cell-cell contacts [65]^.^ Classically, T cells migrate as amoeboid without requiring strong adhesion to extracellular matrix, while they rely on dynamic pseudopods to power their movement [67]. ICOS can act as the Tfh cell pseudopods, which provided dynamics for their migration [66]. ICOS-deficient in Tfh cells will slow down or stop above process, which impaired antigen-specific Ig production due to the decreased adhesion between Tfh cells and GC B cells [68]. Indeed, the more ICOS expressed on Tfh cells, the more sensitive B cells are to antigens [69,70]. Unlike ICOS, as an inhibitory receptor, PD-1 can suppress Tfh cell recruitment, and even restrict their development by antagonizing ICOS and limiting CXCR5 distraction [71]. However, the purpose of PD-1 that plays the side effects on Tfh cells is enforcing a more stringent selection threshold for competing B cells to also promote affinity maturation [71]. Likewise, CD40 is also designed to better promote the antigen-specific antibody production [68]. Profound reduction of mature B cells, reactivities, and antigen-specific antibodies can be observed in the mice with CD40 deficiency genes [72]. In humans, CD40/CD40L signaling deficiency can lead to defective isotype class switching and poor antibody affinity maturation through somatic hyper mutation of B cells [72].

**Table 1:** The manners of Tfh cells supporting B cell functions.

Manners	Molecules/proteins	Effects on B cells programs ^Refs^
Receptor-ligand interactions (cell-cell contacts)	CXCR5, ICOS, PD-1,and CD40	Promoting GCs formation [60,62] Inducing antigen-specific immunoglobulins production [[64], [65], [66]] promoting affinity maturation of antigens-specific antibodies [63] Increasing isotype-switched antibody-secreting B cells [[67], [68], [69]] Enforcing a more stringent selection threshold for competing B cells [71,75]
cytokine secretions (paracrine action)	IL-21, IL-4, and IL-9	Promoting B-cell proliferation [74] Increasing the pools of plasma cells and memory B cells [75,76] Inducing somatic hypermutation in GC B cells [77]

Except for cell-cell contacts, Tfh cells also can provide the helpful signals to B cells thought production of cytokines (paracrine action) [73]. As mentioned above, Tfh cells can secrete various cytokines, most of them are indispensable for B cell programs, such as IL-21, IL-4, and IL-9 [74]. IL-21 can promote B cell proliferation, and is the most potent inducer of PC formation and somatic hypermutation in GC B cells [75]; IL-4 not only can induce Ig isotype switching, but also promote the differentiation of PCs and MBs [76]; IL-9 is mandatory for the development of GC MBs [77].

### 1.3 Aberrant Tfh cell responses account for B cell dysfunction in pediatric RRTIs

**Fig. 3:**
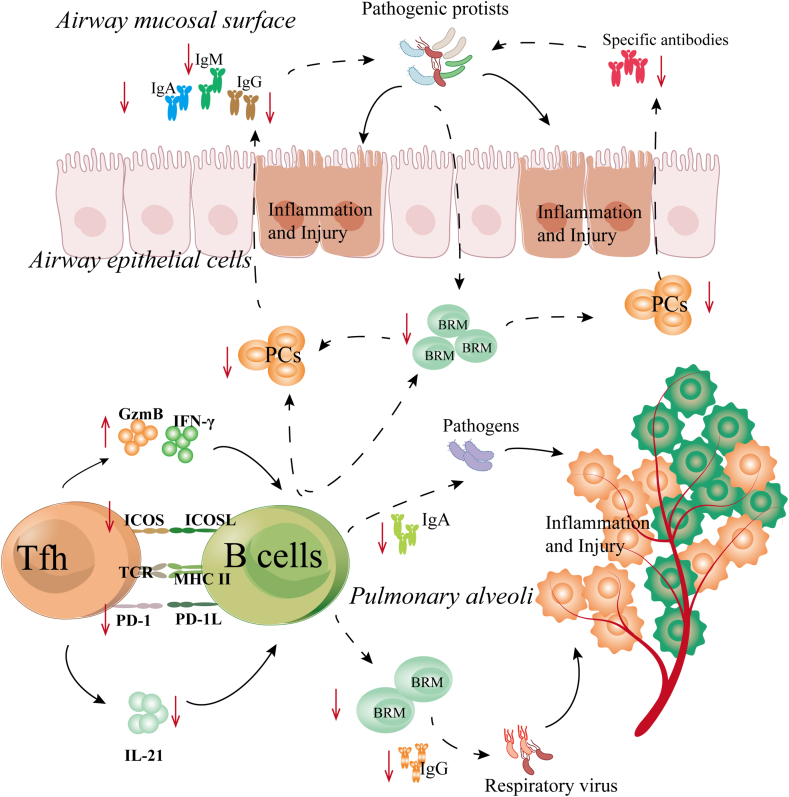
Tfh cells lost the functions of helper B cells in the course of pediatric RRTIs. In disease states, the phenotypes and cytokine secretion of Tfh cells are abnormal, The aberrant phenotypes of Tfh cells can be reflected by the down-regulated expression of CCR7 and ICOS. The abnormal cytokine production of Tfh cells can be presented by up-regulated expression of IFN-γ and GzmB and down-regulated expression of IL-21. However, the phenotypes changed and the abnormal cytokine production of Tfh cells can lead to abrogation of total Ig production, reduction of MB/PC population, and delay of affinity maturation of antigens-specific antibodies. As a results, pathogens will provoke recurrent inflammation and injury of respiratory system, because of humoral immunity deficiency.

As described in the preceding sections, Tfh cells are essential in sustaining B cell dominated humoral immunity, which is necessary for respiratory pathogen clearance. Conversely, aberrant Tfh cell responses accounted for chronic lung diseases development, including asthma, human primary immune deficiencies (PICDs), sarcoidosis, pediatric RRTIs and their corresponding disease models [78]. In the course of pediatric RRTIs, aberrant Tfh cell responses were mainly presented by the reduction of cell frequencies and the expression of a variety of pathogenic cytokines. In addition, the abnormal expression of co-inhibitory and co-stimulatory molecules on Tfh cells is also the reason for their dysfunctions. However, no matter the changes of their phenotypes and frequencies, Tfh cells will lose the functions of helper B cells (Fig. 3). As reported elsewhere, in the children with RRTIs, the following diseases are the most frequent occurrence, nasopharyngitis, nasosinusitis, and tonsillitis [79]. The causes of those diseases were linked with common respiratory pathogen infections, such as RSV, adenovirus, influenza virus, and group A *Streptococcus* (GAS) [80]. Apparently, aberrant Tfh cell responses can be detected in the most of above mentioned diseases. For instance, in the children with GAS-induced recurrent tonsillitis (RT), the abnormity of Tfh cell frequencies and phenotypes has been proved to be the main causes of disease development [11]. Similarly, in the children with RSV provoked lower respiratory tract infections, the aberration Tfh cell-derived cytokine expression was affiliated with RSV reinfections [81]. Noteworthy, while the primary cause of these diseases is abnormalities of humoral immunity, the underlying reason is the dysfunction of Tfh cells in supporting B cell development. Indeed, there is ample evidence suggesting that abnormal Tfh cell responses have a disruptive effect on B cell programs in the course of pediatric RRTIs. In vitro, B cells will present apoptosis and abrogation of total IgA, IgM, and IgG production when they were co-cultured with Tfh cells of children with RRTIs [82]. In vivo, Tfh cells have been found to interfere with the differentiation and development of B cells through multiple pathways. As Jennifer M. Dan and colleagues proposed that Tfh cells from children with RRTIs have three ways to dampen B cell activity, which weakened humor al immune responses and thereby caused pathogen infections/re-infections [11]. At the first level, compared to HC, the lymphoid organs of children with RRTIs contained lower levels of Tfh cells, which reduced the interaction T-B cells in the GCs; Secondly, CD4^+^T cells of children with RRTIs are skewed away from antigen-specific Tfh cell differentiation, which gave rise to the impairment of antigens-specific Igs production; Most importantly, Tfh cells from those patients acquire a killer identity, they can directly kill GC B cells by secreting granzyme B (GzmB) [83]. In general, GzmB is typically secreted by cytotoxic CD8^+^T cells and natural killer cells for killing of target cells [84]. The expression of GzmB by Tfh cells could be counterproductive to the B cell development, which not only impeded the formation of MBs but also induced antibody deficiencies. Besides the changes in phenotype, Tfh cells also exhibited abnormal expression of cytokines in pediatric RRTIs, such as high expression of IFN-γ, while decreased IL-21 secretion. Undoubtedly, the above characteristics of Tfh cells deteriorated the humoral immunity of children with RRTIs [85]. The excessive IFN-γ production by Tfh cells can intervene ig isotype switching of PCs, resulting in the reduction of antigen-specific antibody production after pathogen infections [5]. IL-21 deficiency can decrease B cell proliferation capacity, interfere with GC formation, and inhibit high-affinity antibody production, which exacerbated disease severity upon pathogen reinfection [81].

So far, the underlying mechanisms of aberrant Tfh cell responses are likely to remain unclear in pediatric RRTIs. The over-activation of PI3K signaling pathway is receiving much attention. In CD4^+^T cells, PI3K signaling pathway can be activated by several surface receptors on them, such as TCR, CD28, ICOS, all of them are highly expressed on the Tfh cells [86]. Therefore, PI3K signaling pathway is especially important for the differentiation and activation of Tfh cells. However, hyperactive PI3K signaling in Tfh cells also can induce immune dysregulation in affected patients [81,86]. Apparently, children with RRTIs is among the affected patients. In exploring the pathogenesis of pediatric RRTIs, some studies confirmed that hyperactive PI3K signaling in Tfh cells accounted for disease progression [87,88]. Recently, Julia Bier and colleagues has revealed intrinsic mechanisms that Tfh cells with hyperactive PI3K signaling contribute to B cell dysfunctions [5]. In that research, they proposed that abnormity of surface molecule expression and cytokines production of Tfh cells are the main reasons. Once Tfh cells received hyperactive PI3K signaling, the expression of CCR7 and ICOS was reduced on them, which not only delayed GC formation but also interfered with the T-B interaction in the GCs [89]. Interestingly, as the primary site of PCs and MBs formation, GCs are most influenced by Tfh cells with hyperactive PI3K signaling [90].

## 2 Conclusion

This review summarized the current literatures to highlight the importance of Tfh and B cells in the homeostasis of respiratory system of children, and specifically discussed the abnormalities of their immune responses in pediatric RRTIs (Table 2). In the pathogenesis of pediatric RRTIs, the deficiency of Igs, antigen-specific antibodies and MBs/PCs are responsible for disease progression, all of which are related to Tfh cell phenotypic changes and depletion. However, the mechanisms of Tfh cell dysfunctions in pediatric RRTIs are complex and not fully clarified, which is worthy of further investigation. In recent year, pharmacological interventions that regulate Tfh cell functions have been used in the treatment of pediatric RRTIs, and their clinical efficacy are promising. Noteworthy, while the treatment methods have been optimized, if not properly diagnosis and treatment, RRTIs can seriously affect the growth and development of children. Therefore, it is very important to find out the biomarkers of Tfh cell dysfunctions in the children with RRTIs, which will help to diagnose and treat this disease more efficiently.

**Table 2:** The protectiveness and defects of humoral immunity in the respiratory system.

Status	Effects	Refs
Homeostasis of respirat ory system of children	Inhibiting pathogens invasion by producing immunoglobulins (Igs) Mediating the local defense to pathogens reinfections Alleviating lung inflammation by suppressing pro-inflammatory cytokines production	[14], [15], [16], [17], [18], [19], [20], [21], [22], [23], [24]4
RRTIs in children	Pathogens infections due to mature/naive B cells and Igs deficiency Pathogenic protisits reinfections because plasma cells and memory B cells are immature or/and abnormal	[80], [81], [82], [83], [84], [85], [86], [87], [88], [89], [90]

## Authors' contributions

JD wrote the manuscript. HC and HSL made substantial contributions to figures and tables production. WBC edited and proof read this manuscript. All authors read and approved the final manuscript. The final version of the manuscript was approved by all authors.

## Author contribution statement

All authors listed have significantly contributed to the development and the writing of this article.

## Funding statement

This work was supported by the 10.13039/501100001809National Natural Science Foundation of China (Grant no. 82305313).

## Data availability statement

No data was used for the research described in the article.

## Declaration of competing interest

The authors declare that they have no known competing financial interests or personal relationships that could have appeared to influence the work reported in this paper.
